# Bibliometric Studies and Worldwide Research Trends on Global Health

**DOI:** 10.3390/ijerph17165748

**Published:** 2020-08-09

**Authors:** Esther Salmerón-Manzano, Francisco Manzano-Agugliaro

**Affiliations:** 1 Faculty of Law, Universidad Internacional de La Rioja (UNIR), Av. de la Paz, 137, 26006 Logroño, Spain; esther.salmeron@unir.net; 2 Department of Engineering, University of Almeria, ceiA3, 04120 Almeria, Spain

**Keywords:** COVID-19, asthma, pulmonary disease, HIV/AIDS, diabetes, medicinal plants, musculoskeletal risks, obesity, microplastics, climate change, wastewater treatment, patents, social networks

## Abstract

Global health, conceived as a discipline, aims to train, research and respond to problems of a transboundary nature in order to improve health and health equity at the global level. The current worldwide situation is ruled by globalization, and therefore the concept of global health involves not only health-related issues but also those related to the environment and climate change. Therefore, in this Special Issue, the problems related to global health have been addressed from a bibliometric approach in four main areas: environmental issues, diseases, health, education and society.

## 1. Introduction

Science aims to answer questions, and from a pragmatic approach, science can be understood as a resolution of problems in our society. Science cannot be considered an independent activity, and therefore, it must be remembered that prior studies have been carried out in any given scientific field. Combining scientific aspects with documental aspects gives rise to a certain type of scientific work: scientometric, bibliometric, and informetric studies. These take different titles according to the final approach of the work, such as the following: examining the scholarly literature, evolution, and new trends; worldwide research trends; mapping of the knowledge base; visualizing the knowledge structure; analysis of global research; publication trends; knowledge domain visualization. Scientific literature is losing its relevance more and more quickly, but the aging of literature is not uniform for all scientific subjects. This means that being up to date in a scientific field requires bibliometric studies through which new trends are revealed when undertaking scientific studies of interest to the community. Two topics of special interest to society today are environmental research and public health, and within these larger topics are sub-topics related to global health. Global health, in a broad context, refers to improving health worldwide, reducing disparities, and protecting against global threats that do not consider national borders. This Special Issue aims to provide a global view of all of these global health issues, and through bibliometric studies, we believe that this objective can be achieved. Therefore, articles reviewing the state of the art in any of these fields, bibliometric or scientometric studies, and research articles dealing with a global perspective are welcome.

## 2. Publications Statistics

The summary of the call for papers for this Special Issue on the 28 manuscripts submitted: rejected (12; 43%) and published (16; 67%).

The submitted manuscripts come from many countries and are summarized in Table 1. For this statistic only the first affiliation of the authors has been considered, in which it gives the opportunity to observe 66 authors from nine countries. Note that it is common for a manuscript to be signed by more than one author and for authors to belong to different affiliations. The average number of authors per published manuscript in this Special Issue was seven authors.

## 3. Authors’ Affiliations

There are 23 different affiliations of the authors. Note that only the first affiliation per author has been considered. Table 2 summarizes the authors and their first affiliations.

## 4. Topics

Table 3 summarizes the research conducted by the authors in this Special Issue, by identifying the areas to which they report. It was noted that they have been grouped into four main lines of research: environmental issues, diseases, health and society. They have mainly explored the issue of disease research, these have been: COVID-19, asthma, pulmonary disease, HIV/AIDS, and diabetes. Related to health, they were: medicinal plants, musculoskeletal risks, and obesity. The environmental issues were related to: microplastics, climate change, and wastewater treatment. Finally, research related to education and society: academic performance, patents, bibliometric analysis, and social networks and young people.

## Figures and Tables

**Table 1:** Statistics of authors by country.

Country	Authors
Spain	20
China	18
Vietnam	6
USA	2
Singapore	2
Canada	1
Sweden	1
Australia	2
UK	1
Total	53

**Table 2:** Authors and affiliations.

Author	First Affiliation	References
Martinez-Perez, C.	Universidad Europea de Madrid	[1]
Alvarez-Peregrina, C.	Universidad Europea de Madrid	[1]
Villa-Collar, C.	Universidad Europea de Madrid	[1]
Sánchez-Tena, M. Á.	Universidad Europea de Madrid	[1]
Li, F.	China Medical University	[2]
Zhou, H.	China Medical University	[2]
Huang, D. S.	China Medical University	[2]
Guan, P.	China Medical University	[2]
Hoang, M. T.	Duy Tan University	[3]
Le, H. T.	Hanoi Medical University	[3]
Cascajares, M.	University of Almería	[4]
Alcayde, A.	University of Almería	[4]
Pham, H. Q.	Duy Tan University	[5]
Salmerón-Manzano, E.	University of Almería	[6]
Vu, G. V.	Hanoi Medical University	[7]
Nguyen, C. T.	Duy Tan University	[7]
Wang, M.	Chengdu University of Technology	[8]
Liu, P.	Chengdu University of Technology	[8]
Zhang, R.	Chengdu University of Technology	[8]
Li, Z.	Chengdu University of Technology	[8]
Li, X.	Chengdu University of Technology	[8]
Hall, B. J.	Johns Hopkins University	[9]
Hoang, C. L.	Nguyen Tat Thanh University	[9]
McIntyre, R. S.	University of Toronto	[10]
Pham, N. M.	Curtin University	[11]
Vu, H. T. T.	Hanoi Medical University	[11]
Nguyen, H. T.	National Institute of Nutrition	[11]
Esteban-García, B.	University of Almería	[12]
Agüera, A.	University of Almería	[12]
Sánchez-Pérez, J. A.	University of Almería	[12]
Nghiem, S.	Griffith University	[13]
Afoakwah, C.	Griffith University	[13]
Doan, L.P.	Nguyen Tat Thanh University	[13]
Aparicio-Martinez, P.	University of Cordoba	[14]
Perea-Moreno, A. J.	University of Cordoba	[14]
Martinez-Jimenez, M. P.	University of Cordoba	[14]
Redel-Macías, M. D.	University of Cordoba	[14]
Vaquero-Abellan, M.	University of Cordoba	[14]
Pagliari, C.	University of Edinburgh	[14]
Gómez-Galán, M.	University of Almería	[15]
Callejón-Ferre, Á. J.	University of Almería	[15]
Pérez-Alonso, J.	University of Almería	[15]
Díaz-Pérez, M.	University of Almería	[15]
Carrillo-Castrillo, J. A.	University of Seville	[15]
Qin F.	Dalian University of Technology Library	[16]
Du J.	Liaoning Ocean and Fisheries Science Research Institute	[16]
Gao J.	Dalian University of Technology Library	[16]
Liu G.	Liaoning Ocean and Fisheries Science Research Institute	[16]
Song Y.	Liaoning Ocean and Fisheries Science Research Institute	[16]
Yang, A.	Technology Center of Dalian Customs District	[16]
Wang H.	Ocean University of China	[16]
Ding, Y.	Dalian University of Technology Library	[16]
Wang Q.	Ocean University of China	[16]
Tran, B. X.	Hanoi Medical University	[3,5,7,9,10,11,13]
Ha, G. H.	Duy Tan University	[3,5,7,9,10,11,13]
Nguyen, L. H.	Vietnam National University	[3,11]
Vu, G. T.	Nguyen Tat Thanh University	[3,5,7]
Latkin C. A.	Johns Hopkins University	[3,5,7,9,10,11,13]
Ho, C.S.H.	National University Hospital	[3,5,7,9,10,11,13]
Ho, R.	National University of Singapore	[3,5,7,9,10,11,13]
Phan, H. T.	Hanoi Medical University	[5,9,10]
Vu, G. T.	Nguyen Tat Thanh University	[5,9,10]
Pham, H. Q.	Duy Tan University	[7,10,11,13]
Nguyen, T.P.	Nguyen Tat Thanh University	[11,13]
Garrido-Cardenas, J. A.	University of Almería	[4,6,12]
Manzano-Agugliaro, F.	University of Almería	[4,6,12]

**Table 3:** Topics for worldwide research trends on global health.

Bibliometric Studies	Number of Manuscripts	References
Environmental issues	3	[2,12,16]
Diseases	6	[3,5,7,9,10,11]
Health	3	[6,13,15]
Education and society	4	[1,4,8,14]
